# Discovery and characterization of anti-cancer peptides from a random peptide library

**DOI:** 10.1371/journal.pone.0293072

**Published:** 2024-02-13

**Authors:** Pavan Kumar Puvvula, Anne M. Moon

**Affiliations:** 1 Department of Molecular and Functional Genomics, Weis Center for Research, Geisinger Clinic, Danville, Pennsylvania, United States of America; 2 Department of Human Genetics, University of Utah, Salt Lake City, Utah, United States of America; 3 The Mindich Child Health and Development Institute, Hess Center for Science and Medicine at Mount Sinai, New York, New York, United States of America; University of South Florida, UNITED STATES

## Abstract

We performed a forward genetic screen to discover peptides that specifically target breast cancer cells using a Penetratin tagged, random 15mer peptide library. We identified a group of novel peptides that specifically inhibited the proliferation and survival of breast cancer cells without affecting normal primary mammary epithelial cells or fibroblasts. The intrinsic apoptotic pathway is activated by these peptides in the face of abnormal expression of numerous cell cycle regulatory genes. Associated alterations in histone marks, nuclear structure, and levels of critical RNA binding proteins vary in a peptide specific manner. This study demonstrates a novel method for the discovery of new potential therapeutic peptides.

## Introduction

Cell-penetrating peptides (CPPs) are comprised of amino acids that function as carrier molecules to transduce various cargo molecules across cell membranes [1–4]. In the recent past, several potential anti-cancer therapeutic peptides have come to light utilizing the advantages of CPPs. The main principle underlying these discoveries is the identification of dominant-negative domains of proteins with anti-cancer properties. By implementing this methodology, MYC and ATF5-derived CPPs were shown to restrict tumor growth and have now progressed to clinical trials [5–11]. In line with the above studies, dominant-negative domains of ANXA1, p50, connexin-43, and Grb-7 were shown to inhibit gastric and colon cancers [12], breast cancer [13], glioma [14], and human epidermoid carcinoma [15]. The U.S. Food Drug Administration has authorized 15 peptide-based drugs in recent years [16].

One of the main challenges in developing an effective peptide is identifying a domain that can disrupt cancer-specific cellular functions but not harm normal cell types. The next challenge is to enhance the anti-cancer potential of the effective region by chemical alterations or addition of other moieties. Such modifications have been shown to generate peptides that function more effectively than the wild-type domain for MYC and ATM [7]. The time- and effort- and resource- intensive nature of these challenges hinders preclinical studies and progress toward clinical implementation. Thus, there is continued reliance on nucleic acid (siRNA, shRNA, CRISPR) or pharmacological (such as kinase inhibitors) treatments despite their significant side effects.

Forward genetic approaches have been successfully employed in modern biology to discover critical molecular players and signaling pathways [17]. A well-established procedure is to randomly overexpress a population of genetic elements in a target cell type and screen for the desired phenotype, then recover the causative construct and characterize the functional properties that drive its efficacy. This general approach offers a potentially effective strategy for us to identify novel peptides that can inhibit cancer cell proliferation and survival through the use of random peptide libraries.

Random peptide libraries contain an enormous number of peptides that can theoretically bind to a huge variety of target molecules. Therefore, incorporating random peptide libraries with phage display allows one to isolate peptides that bind to cell surface receptors, enzymes, tumor antigens or protein-interactors [18, 19] Similarly, CPPs with high intracellular translocation efficiency were discovered by viral introduction of a random peptide library with virus *in vitro* [20]. In line with the above studies, tumor lineage-homing CPPs were identified from random peptide libraries constructed using mRNA display technology [21]. These studies highlight the importance of random peptide libraries in discovering novel molecules.

We previously demonstrated that RNA-binding domains of hnRNPK [22], hnRNPU [3], and RBM39 [23] act as dominant-negatives and reduce cancer cell growth and viability. However, identifying dominant-negative domains of potential therapeutic target proteins is a laborious process with no guarantee that they will have the desired cellular effect. Here we exploit a forward genetic screen of a random peptide library to identify novel cell penetrating peptides with anti-cancer properties.

We generated and screened a random DNA library encoding CPP-tagged 15mer peptides and screened for clones that disrupt the growth and survival of MDA-MB231 triple negative breast cancer cells but not of two normal cell types. We further evaluated their effects on apoptosis, gene expression, epigenetic marks, and levels of critical factors such as Lamins, spliceosomal and other RNA binding proteins. Our results demonstrate the applicability and efficacy of this novel method to identify disease-specific cell-penetrating peptides in an unbiased manner. This approach could provide new avenues for peptide research in basic biology and has potential clinical applications.

## Results

### Generation of the random peptide library

Our recent investigations on RBM39-RRM3-, SAFA-RGG- and hnRNPK-RGG- [3, 22, 23] derived cell-penetrating peptides revealed that treating cells with a penetratin-tagged peptide from these functional domains inhibited cancer cell growth and survival. This mode of action prompted us to develop a new unbiased technique to discover novel cell-penetrating peptides that could be developed therapeutically for use in various disease models.

**Fig 1:**
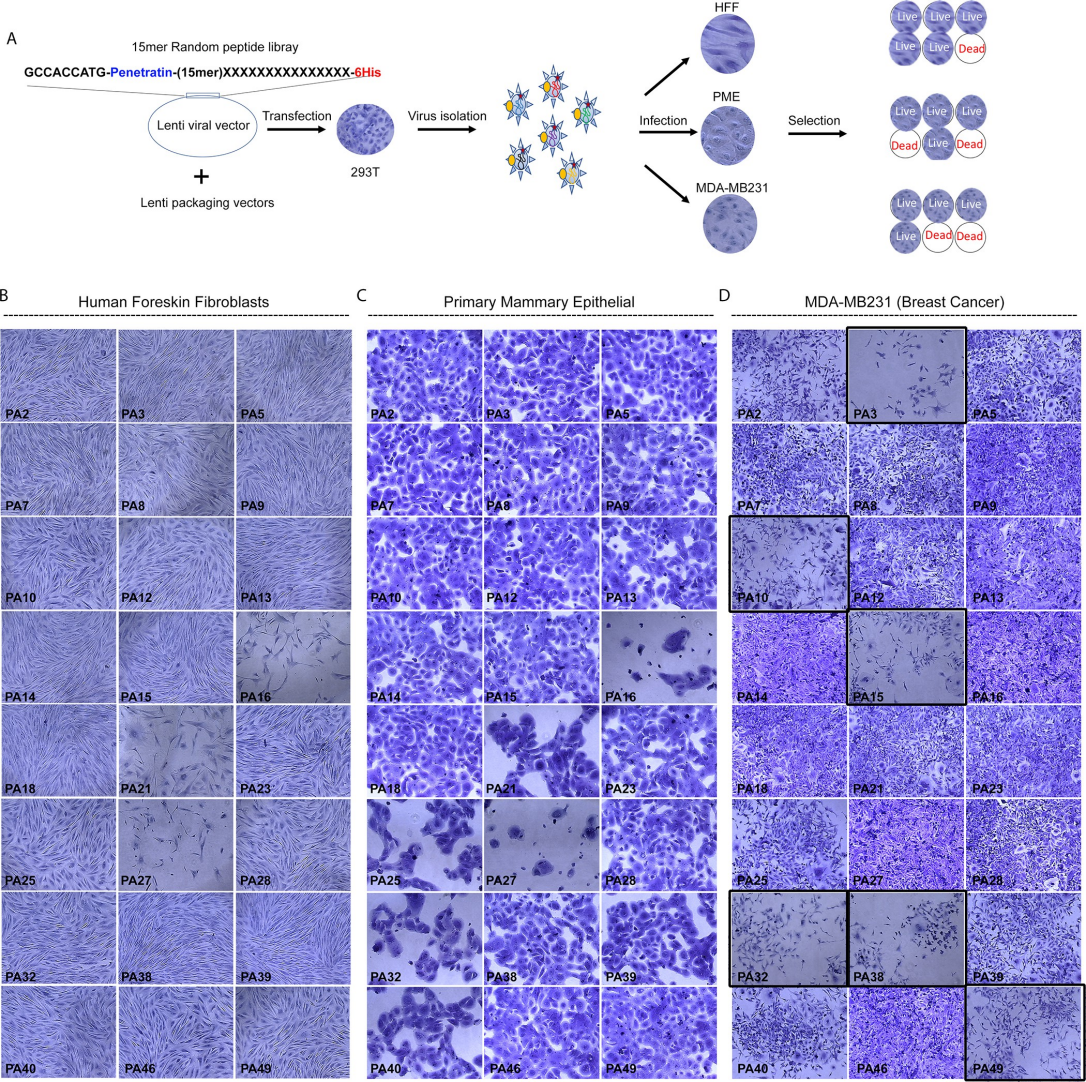
Identification of anti-cancer peptides from a random peptide library. A) Schematic of cloning 15mer random peptide library into pLenti-puro vector and co-transfection with packaging vector into 293T cell to generate viral particles, in turn followed by screening procedure to identify breast cancer cell-specific peptides by transducing HFF, PME and MDA-MB231 cells with lentivirus expressing 15mer random peptides. B-D) Representative light microscopic images of crystal violet-stained HFF, PME and MDA-MB231 cells after 4 days of incubation in Puromycin selection media. Black boxed fields in D highlight peptides with effects specifically in MDA-MB231 breast cancer cells.

We synthesized DNA sequences that contain a Kozak sequence and encode Penetratin, a 15mer random peptide and a 6X His tag. The random 45 nucleotides (which encode 15mer peptides in frame with the upstream Penetratin tag) were synthesized by IDT DNA with 1:1:1:1 ratio of A: T: G: C by the machine-mixing method. These fragments were cloned into a pLenti-puro vector (Fig 1A, schematic). As proof of principle, we sequenced 240 of these and confirmed that all the individual clones harbored a unique 15mer amino acid encoding DNA sequence. S1 Table shows the 15mer amino acid sequences for 78 of these. Among these, 31 contained a stop codon located such that the peptide would likely not be useful, so these were not pursued further. By employing a standard lentivirus production protocol, we isolated lentivirus particles containing the remaining 47 clones (S1 Table, column 3). Since these peptides (P) are artificially (A) designed and derived from random nucleotide library sequences, they are hereafter termed with the prefix PA followed by a numeric identifier. We randomly selected 9 lentiviral particles and tested for their expression in human fibroblasts (HFFs) using immunofluorescence to detect the His tag after 72 hours of incubation in the selection medium. As shown in Supplemental Figure (S1 Fig), this confirmed the presence of the virally produced PA peptides in both cytoplasm and nucleus.

We next tested whether virally expressed peptides would affect the proliferation or survival of cells and if so, set out to identify those peptides that only impact cancer cells. The schematic representation of the cellular screening procedure is shown at the right in Fig 1A. For normal cell types we chose human foreskin fibroblasts (HFFs) and primary mammary epithelial cells (PMEs). The cancer cell line we employed for the screen was the triple-negative B breast cancer line MDA-MB 231. Cells were grown in 24 well plates and transduced with the 47 individual lentivirus particles and incubated for four days in the presence of puromycin selection media. The effects on total cell number were detected using crystal violet staining for viable cells. Results with all 47 peptides are presented in S1 Table. Fig 1B–1D show examples of light microscopy images of 24 wells with each peptide in HFFs, PMEs and MDA-MB231 cells. 17 of the clones did not affect the survival of any of the cell lines (S1 Table, Column D- F, cells highlighted with green). As shown in S1 Table, there were four patterns of response over the three cell lines: 1) lethal to all cell types; 2) toxic to HFFs and/or PMEs as evidenced by reduced cell number and altered morphology (Fig 1B–1C, S1 Table); 3) breast-cell specific (PA25, PA32, PA40 affected both PME and MDA-MB231 cells, Fig 1C and 1D) and 4) effect restricted to MDA-MB231 cancer cells (PA2, PA3, PA10, PA15, PA38, and PA49, Fig 1D, images highlighted with black outline).

To confirm and extend these findings, we tested the effects of PA2, PA3, PA10, PA15, PA38, and PA49 and 18 other lentivirus particles on the survival of PME and MDA-MB231 cells. Virus transduced cells were seeded at the rate of 5000 cells per well in 96 well plates and grown for 3 days in selection medium. Expression of these 6 peptides reduced viability of MDA-MB231 cells but not PMEs as assessed by crystal violet staining (S2A and S2B Fig, red circles in A highlight peptides with differential effects in MDA-MB231 cells) and with the CellTiter-Glo 2.0 cell assay which measures intracellular ATP (S2C Fig, MDA-MB231 cells, red bars denote peptides that were significantly different from control and differentially effective on MDA-MB231 cells in panels A and B). Next, we assayed the effect of these peptides on the growth/survival of MDA-MB231 cells in a time-course analysis. We seeded equal numbers of virally transduced MDA-MB231 cells and measured the total number of cells on days 1, 3, and 5 (S2D Fig): all 6 peptides (red asterisk next to peptide number indicates the 6 differentially active peptides shown in panel A) reduced the number of viable cells in a time-dependent manner.

### Direct treatment of breast cancer lines with synthetic PA peptides decreases cell survival but has no effect on normal-like mammary cell lines

**Fig 2:**
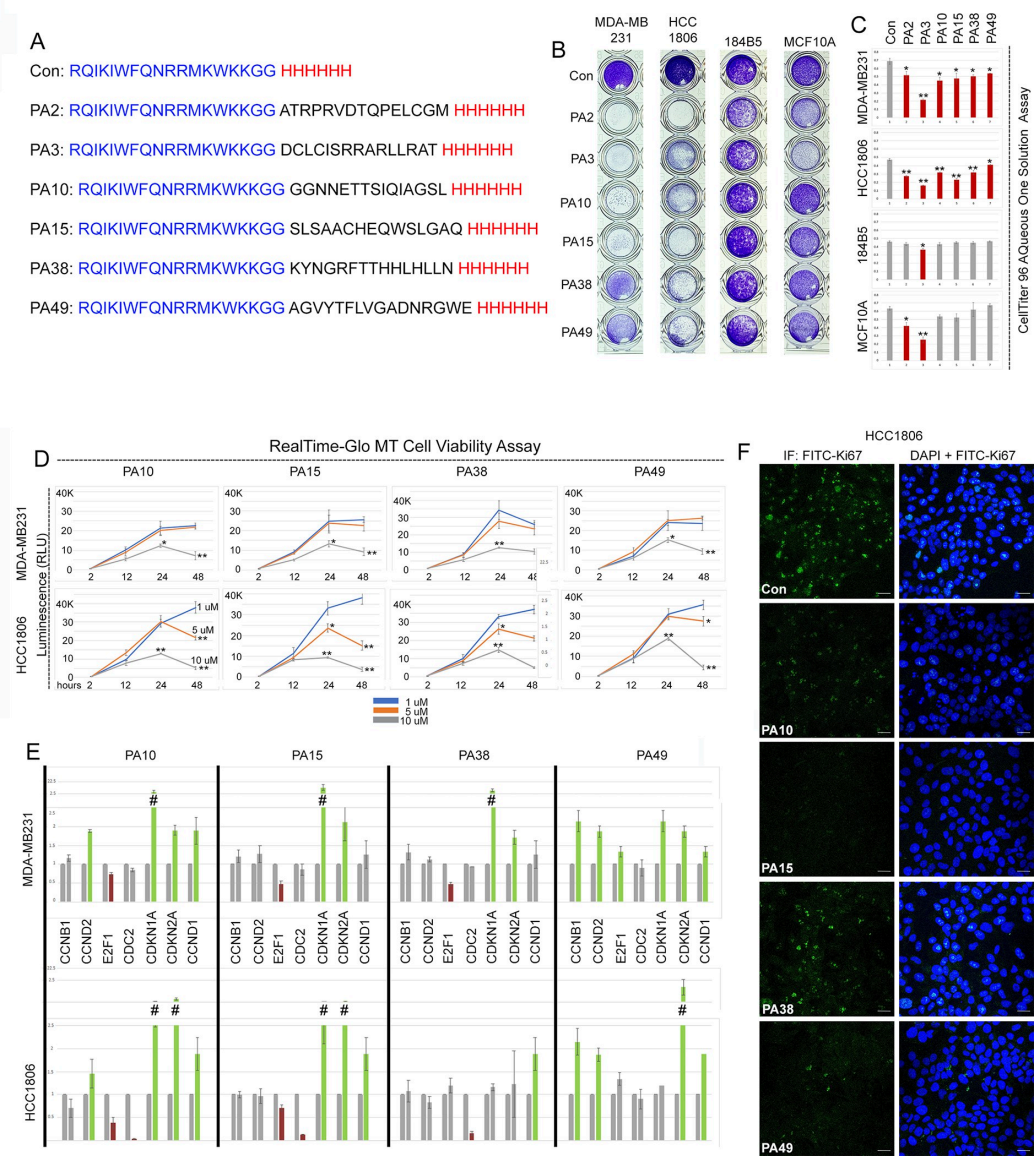
Cell-penetrating 15mer peptides decrease the proliferation and viability of cancer cells. A) Amino acid sequences of the cell-penetrating peptides negative control (Con), PA2, PA3, PA10, PA15, PA38 and PA49. The sequence of Penetratin is in green, His tag in red and 15mer peptides are in black. B) Representative light microscopic images of crystal violet-stained MDA-MB231, HCC1806, 184B5 and MCF10A cells treated for 24hr with 10 μM concentration of 15mer peptides (listed at left). C) Quantification of absorbance (490) of MDA-MB231, HCC1806, MCF10A, and 184B5 cells after 24hr of peptide treatment. Absorbance values (Y-axis) indicate the number of viable cells which reflect altered cell proliferation and/or survival as assayed by the Cell Titer 96 Aqueous One Solution Cell Proliferation Assay. Peptide treatments are labeled on X-axis. * indicates p<0.05 and ** indicates p<0.01 relative to control. D) Quantification of luminescence (RLU, proportional to living cell number) of peptide treated cells with the RealTime-Glo MT Cell Viability assay. Y-axis indicates the relative luminescence units; X-axis indicates the time of incubation in hours. * indicates p<0.05 and ** indicates p<0.01 relative to control. E) qRT-PCR analysis of cell cycle genes in total RNA isolated after 24hr of peptide treatment. Green and red bars indicate significantly up and down-regulated relative to control, respectively. Hashtags (#) represent gaps in graph to accommodate for scale required for high levels of some transcripts. F) Staining for Ki67^+^ cells after 24hr of peptide treatment in HCC1806 cells. Scale bar, 50 μm. Quantitative results are presented in S2 Table.

We next investigated the effect of directly treating cells with the peptides (as opposed to virally producing them) on both MDA-MB231 cells and an additional triple-negative B breast cancer cell line (HCC1806), as well as two nonmalignant, transformed breast lines: MCF10A and 184B5. We synthesized the six peptides with MDA-MB231 specific action (Fig 2A and 2B). Previously we showed that Penetratin-tagged synthetic peptides derived from known domains of three different RNA binding proteins efficiently enter T47D and MDA-MB231 breast cancer cells [3, 22, 23]. Here we examined penetration of these 6 novel random peptides into HCC1806 cells treated with 10μM peptide for 4 hours followed by immunofluorescence assay for the His tag (S3A Fig). All six were present in the cytoplasm with varying levels of nuclear penetration; in particular, PA49 had no detectable signal in the nucleus. PA2 and PA49 were also present in cytoplasmic/membrane aggregates.

We tested the effects of treatment at 10μM concentration for 24 hours on the total cell number of MDA-MB231, HCC1806, MCF10A and 184B5 cells using crystal violet staining (Fig 2B) and as quantitated with the CellTiter 96 Aqueous assay (Fig 2C). Consistent with the results obtained in the initial screen, PA10, PA15, PA38, and PA49 did not affect the growth of the normal-like MCF10A and 184B5 cells. However, in contrast to the result with virally expressed peptide, direct treatment with PA2 and PA3 reduced the number of both normal-like cell types, so they were not studied further. This experiment was also performed at 48 hours with similar findings (S2 Table). Analysis of the IC50 curves for viability at 48 hours (S2 Fig) which showed a consistent IC50 for all 4 test peptides at ~10 μM.

We assayed the peptides’ effect on cancer cell viability over real time at 3 different peptide concentrations (1, 5 and 10 μM, Fig 2D). At 1 μM concentration, neither cancer cell line responded to any of the peptides (Fig 2D, blue lines in each graph). At 5 μM (orange graph lines), HCC1806 cells showed some decreased viability at 24 hours (Fig 2D, lower row graphs), while MDA-MB231 cells were not affected (Fig 2D, upper row graphs). As anticipated, at 10 μM concentration, all four peptides decreased the cell number in both lines (Fig 2D, gray lines).

In addition to effects on viability, we observed altered growth patterns in surviving cells with prolonged doubling times (not shown) leading us to question whether the peptides disrupted pro-proliferative gene expression. Treatment with 10 μM peptide for 24 hours followed by isolation of RNA and qRT-PCR analysis showed that levels of transcripts for cell cycle genes *CCNB1*, *CCND2*, *E2F1*, *CDC2*, *CDKN1A*, *CDKN2A* and *CCND1* were similarly affected in the 2 different cell lines. Statistically significant increases in the levels of transcripts for *CDKN1A* and *CDKN2A* (encoding the antiproliferation/proapoptotic proteins p21 and p16/p14, respectively) were observed with all peptides but PA38 in HCC1806 cells. With the exception of PA49, the levels of transcripts encoding the pro-proliferative transcription factor E2F1 were decreased. The effects on the other transcripts were more variable showing modest cell- and peptide- specific responses (Fig 2E).

We then used immunofluorescence to assay for Ki67+ proliferating cells after treatment with 10μm peptide for 24 hours. Consistent with the gene expression results, all four peptides resulted in a reduced number of Ki-67+ cells relative to control in both cell lines (HCC1806 cells are shown in Fig 2F and MDA-MB231 in S3B Fig; quantitated in S3 Table). These cumulative results show that PA peptides influence cancer cell growth by decreasing both viability and proliferation.

### Treatment of breast cancer cells with PA peptides increases apoptosis by activating Caspases and expression of genes in both the intrinsic and extrinsic apoptotic pathways

**Fig 3:**
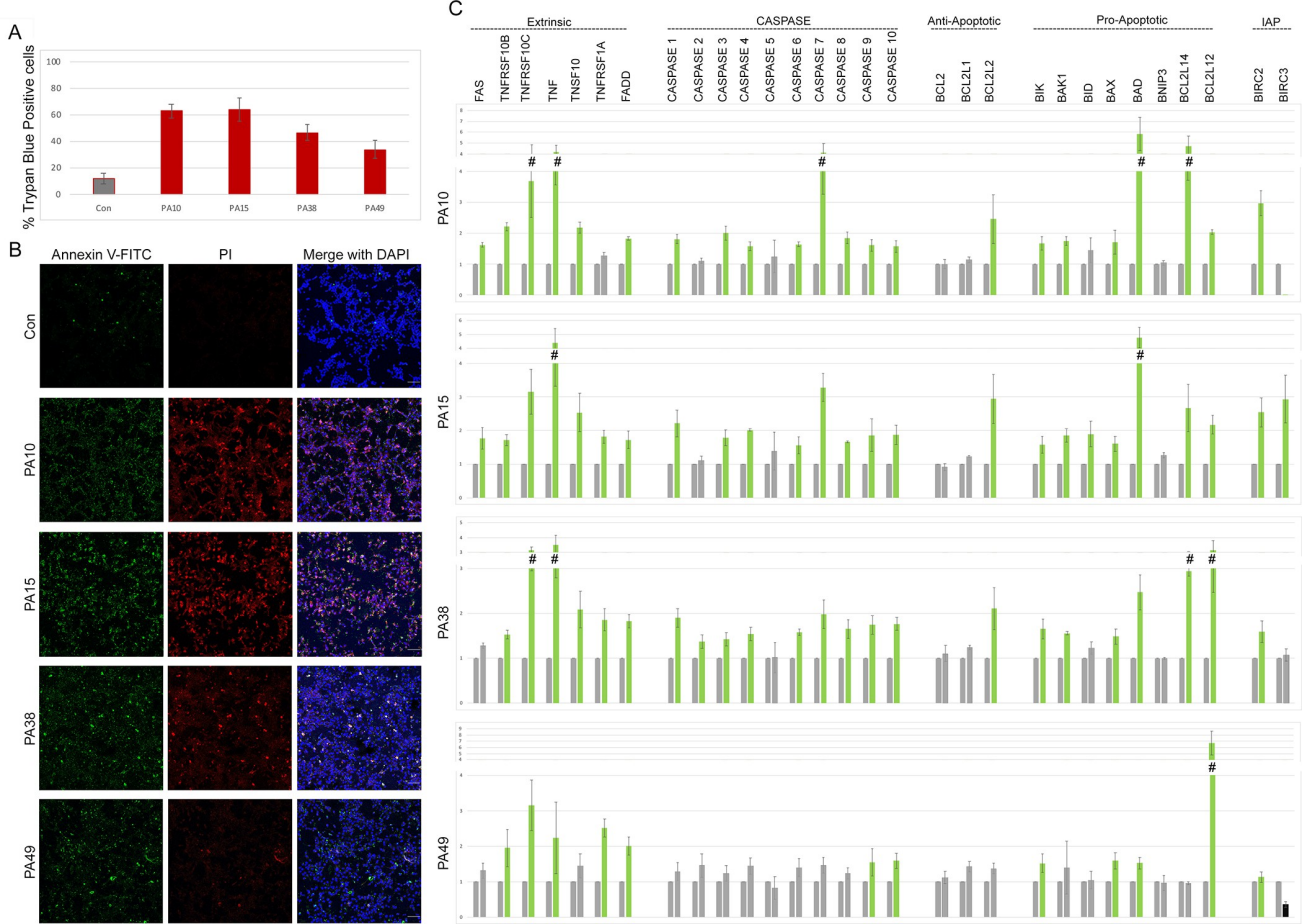
Cell-penetrating 15mer peptides induce apoptosis and necrosis by activating apoptotic transcription pathway genes. A) Quantification of total dead HCC1806 breast cancer cells using Trypan Blue staining after 24 hr of 10 μM peptide treatment. * indicates p<0.05 and ** indicates p<0.01 relative to control. B) Representative immunofluorescence images of Annexin V and Propidium Iodide stained HCC1806 cells after 24hr of peptide treatment. Individual channels (Hoechst = Blue, Annexin V = Green, PI = Red) and merged images are shown for each treatment. Scale bar, 50 μm. * indicates p<0.05 and ** indicates p<0.01 relative to control. Peptide treatments are noted at the left of the panels. C) qRT-PCR analysis of apoptotic pathway genes in total RNA isolated from HCC1806 after 24hr of the PA10, PA15, PA38 and PA49 peptide treatments. Transcripts under investigation are indicated at top.

Peptide-mediated effects on total cell number and on cell proliferation (Ki67) led us to assay the amount of peptide-induced cell death using trypan blue inclusion/exclusion. After 24 hours of 10μM peptide treatment all four peptides killed a significantly greater number of HCC1806 cells than control peptide (Fig 3A). Consistent with this finding was increased Annexin V staining, which labels early apoptotic cells (FITC, Fig 3B, quantitated in S4 Table) and propidium iodide staining, which labels both late apoptotic and necrotic cells (red, Fig 3B, quantitated in S5 Table). PA10 and PA15 induced the greatest number of Annexin V and PI positive cells while there was little detectable PI staining seen in PA49 treated cells.

Apoptosis is controlled by the balance between pro and anti-apoptotic signaling and gene expression. To identify apoptotic transcriptional responses to peptide treatment, we selected 30 genes linked to apoptosis (grouped in 5 classes based on pathway) and assayed their transcript levels in HCC1806 cells (Fig 3C). Green bars show significantly upregulated genes. The profiles for PA10, PA15 and PA38 are very comparable. The response to PA49 differed from the other 3 peptides in that the levels of most *CASPASE* transcripts were not increased by the peptide despite apoptosis being the primary means of cell death as detected with the Annexin V assay.

To assay for activated Caspase proteins as the effectors of apoptosis, we assayed peptide treated HCC1806 cells with antibodies to cleaved Caspase 3, 7 and 9. Compared with control, all 4 peptides increased the signal of cleaved Caspase 3 staining in the cytoplasm and PA10, PA15 and PA49 also had increased signal for cleaved Caspase 7 (S5A and S5B Fig). We also observed PARP signals in the nucleus of cells treated with PA15 and PA49 (S5C Fig), which indicates DNA damage/genotoxic stress [24]. We also used western blot to assay for activated Caspase 3 and 8 and for cleaved PARP (S6 Fig). This showed an increase in cleaved Caspase 3 and PARP with all four peptide treatments as compared with control peptide indicating activation of the intrinsic apoptotic cascade. No cleaved Caspase 8 was detected in any of the treatments. Combined with the Annexin V and propidium iodide assays, the data indicate that both apoptosis and likely some degree of necrosis contribute to cell death in PA10, PA15 and PA38 treated cells while there was little evidence of necrosis in PA49 treated cells.

### PA peptides alter the levels and localization of nuclear structural proteins and histone marks

**Fig 4:**
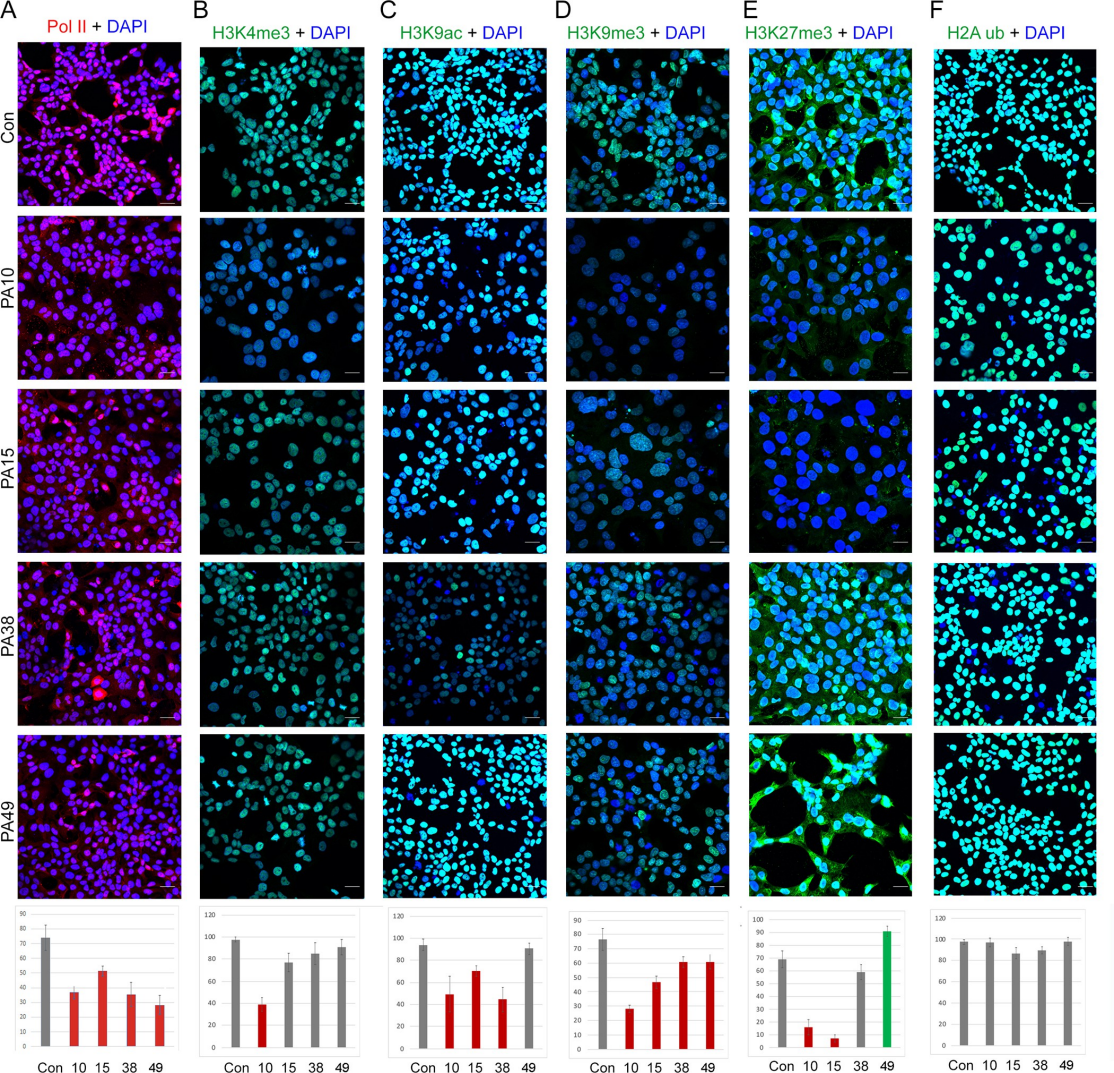
Cell-penetrating 15mer peptides alter the epigenetic marks in HCC1806 cells. A-F Immunofluorescence analysis of Pol II, H3K4me, H3K9ac, H3K9me3, H3K27me3, and H2AK119ub, marks in HCC1806 cells treated with PA10, PA15, PA38 and PA49 peptides. Scale bar, 10 μm. Graphs at bottom show quantification of % cells positive for the indicated histone epigenetic marks in response to the peptide treatments. * indicates p<0.05 and ** indicates p<0.01 relative to control.

We sought to further understand the mechanism of peptide action leading to altered gene expression and thus characterized the global effect of peptides on nuclear structure and function-related proteins such as Pol II, Lamin A/C, Lamin B1 and PML proteins. As shown in Fig 4A, immunofluorescence analysis revealed that all four peptides diminished the intensity of Pol II signals in the nuclei of HCC1806 cells compared to negative control peptide (quantitated at bottom). PA10, PA15 and PA38 markedly reduced Lamin B1 and Lamin A/C protein levels (S7A and S7B Fig) while effects of PA49 were minimal on these two proteins. Lamin B1 staining in PA15 treated cells also revealed wrinkled nuclear envelopes with distorted nuclei and herniation of the envelope compared to the control cells. Despite these findings, we did not detect a significant effect on PML bodies with any of the peptides (S7C Fig). Chromatin structure and epigenetic marks play a significant role in both transcription [25] and nuclear organization [26]. The peptides’ effects on Pol II occupancy and nuclear envelope structure suggest that they disrupt higher order chromatin structure. We assayed H3K4me3, H3K9ac, H3K9me, H3K27me3 and H2Aub histone marks after peptide treatment of HCC1806 cells. As anticipated, all 4 peptides affected histone epigenetic marks (Fig 4A–4F, graphs at bottom quantitate the percent of mark-positive cells for each peptide) however, there was not one pattern of response that would predict the consistent loss of Poll II occupancy we observed with all four peptides (i.e., no net “repressive” histone mark pattern). Nonetheless, all together these observations suggest that there are pervasive changes in nuclear structure and dysregulation of histone marks that contribute to the effects of all four peptides on gene expression and the survival of breast cancer cells.

### PA peptides alter the levels of RNA binding proteins with diverse, critical functions

Emerging evidence shows that chromatin-associated RBPs have regulatory functions in both transcription, splicing and epigenetic regulation [27]. Thus, we tested the consequence of peptide treatment on the levels and localization of some RBPs relevant to cancer cell proliferation and survival [28–34]. HCC1806 cells were treated with 10μM peptide for 24 hours and subjected to immunofluorescence analysis using the antibodies shown in S4 Fig. SC35 plays an instrumental role in nuclear speckle formation and function; its loss induces genomic instability and cell cycle arrest [35] likely attributable, at least in part, to widespread effects on splicing and transcription [36, 37]. Surprisingly, levels of SC35 were increased in response to PA10 and PA38 (S4A Fig) and decreased in PA15 treated cells. All four peptides decreased the size and number of DDX21 puncta (best visualized in the digitally zoomed insets in the lower left corner of each panel in S4B Fig); this protein plays important roles in ribosomal RNA biogenesis and ribosome assembly, RNA editing, RNA transport, and transcription. PA10, PA15 and PA49 markedly reduced the signal intensity for the pre-mRNA processing and spliceosome nucleator hnRNPC1, whereas PA38 increased it (S4C Fig). In contrast, while hnRNPK also functions in a wide range of RNA processing functions in the nucleus and in the cytoplasm, it was only affected in PA15 treated cells which had an increase in both nuclear and cytoplasmic signal (S4D Fig). Peptide treatments had no effect on the amount or location of DDX3, RBM39 or hnRNPU (data not shown). These data show that each peptide has a discrete “profile” of effects on specific RNA binding proteins and would differentially impact critical RNA-regulated processes.

## Discussion

We recently demonstrated that RNA-binding domains of hnRNPU, RBM39 and hnRNPK [3, 22, 23] inhibit the survival of a range of cancer cells. Here we established an unbiased method to discover peptides with anti-cancer properties. Previously, several studies have explored the benefits of random peptide libraries with the phage display technique [19, 38–41] to identify cancer cell-specific ligands [42–45]. The main drawback in these studies was the lack of a tag or peptide to facilitate cargo penetration and intracellular distribution; here we employed the Penetratin tag to overcome this issue. Our results show that a subset of the random peptides we generated disrupt breast cancer cell proliferation and survival without affecting normal cells. Our data indicate that these effects stem from impacts on multiple critical cellular processes including dysregulation of histone marks, altered cell cycle and cell death regulatory gene expression, disrupted nuclear membrane structure, and abnormalities in the amount and location of a subset of RNA binding proteins with known effects on cancer cell survival. We found evidence of activation of the intrinsic apoptotic pathway with all 4 peptides and the particular combination of molecular and cellular responses observed in response to each peptide varied, indicating that their molecular targets are different and specific. The concept of targeted efficacy is further supported by the lack of effect on normal cell types.

Several groups have demonstrated a correlation between histone mark alterations and cancer severity [46–50]. Consistent with this, levels of H3K9ac marks were markedly reduced by PA10, PA15, and PA38 while all four peptides impacted H3K9me3 and Pol II occupancy. Such findings would predict widespread transcriptional consequence beyond those we specifically assayed here. Similarly, SC35, DDX21 and hnRNPC1 are important RBPs whose quantity and/or localization are impacted in a peptide-specific manner. These results here are consistent with previous reports that abrogation of DDX21 [51] and hnRNPC1 [28] function inhibits cancer cell proliferation and survival. Abnormal quantities of nuclear structural proteins such as Lamins in peptide-treated cancer cells indicate the broad spectrum of the peptides’ action as these can be both a contributor to, and an effect of, transcriptional changes. It is worth noting that PA49 had minimal detectable effect on the nuclear lamina proteins we assayed, and this peptide did not penetrate the nucleus (S2A Fig); this peptide also had the least pronounced effects in the majority of assays, with the exception of Ki67 positive cell number (S3 Table).

We did not detect significant stretches of peptide sequence homology with known human proteins leading to the question of the molecular targets and mechanism of action of PA peptides, which are beyond the scope of this study. Even without this information, our forward approach could be applied to any cancer cell type and potentially allow discovery of disease-specific peptides that can then be tested in preclinical studies. Because of their small size, the peptides show excellent stability, likely by escaping protease cleavage. The concentrations and stability of the peptide in lysates or cells can be tested based on the His signals. Further the His-tag can be exploited for conducting fast and sensitive mass-spectroscopy analysis or pull-down experiments to identify the cellular factors bound by a given peptide.

In total our work establishes a method with utility in discovering novel anti-cancer peptides as an alternative to existing drug discovery technology. The peptides may be comparable to other anti-tumor peptides such as OmoMYC and d/n/ATF5-2 CPPs [52–54]. Future *in vivo* testing of the peptides is needed to further develop these peptides and establish their anti-cancer properties in hopes of developing therapies that could decrease the need for more toxic chemotherapeutic approaches.

### Materials and methods

Cell Culture: MDA-MB231, HCC1806, HFF1, MCF10A, PME and 184b5 were obtained and maintained as per the procedures prescribed by ATCC [23].

Cloning: DNA library encoding 15mer random peptides encoding was synthesized by IDT DNA technology with over hangs of the BamH1 and EcoR1. These fragments were cloned in BamH1/EcoR1 digested pLenti-puro vector. Individual plasmids were isolated from single stable bacterial colonies and used for transfection studies. All the sequences were confirmed.

FP: GATCC GCCACCATG

CgccagattaaaatttggtttcagaaccgccgcatgaaatggaaaaaaggcggcNNNNNNNNNNNNNNNNNNNNNNNNNNNNNNNNNNNNNNNNNNNNNcatcaccatcaccatcactgaG

RP: AATTC tcagtgatggtgatggtgatgNNNNNNNNNNNNNNNNNNNNNNNNNNNNNNNNNNNNNNNNNNNNNgccgccttttttccatttcatgcggcggttctgaaaccaaattttaatctggcgCATGGTGGCG

pLenti-puro was a gift from Ie-Ming Shih (Addgene plasmid # 39481) [55]

psPAX1 was a gift from Didier Trono (Addgene plasmid # 12260)

pMD2.G was a gift from Didier Trono (Addgene plasmid # 12259)

Briefly, lentivirus was produced by transfection of 10 μg of plenti-puro-15mer-PA clone, 10 μg of psPAX and 5 μg of VSVG plasmids into 293T cells by TurboFectin 8.0 transfection reagent at the ration of 1:3 (DNA: Reagent) as per the manufacturer’s protocol. Viral supernatant was collected after 48 hr of the transfection and filtered through 0.45-μM filters. HFFs, PME, MDA-MB-231, MCF10A, 184B5 cells were transduced with their complete growth medium containing polybrene (8 mM) and 500 μl of 15mer PA encoding lentivirus. After one day of infection, growth media was replenished with the puromycin selection media. Cells were cultured for the indicated days in the figures and subjected to follow-up studies.

Antibodies: H3K9me3 (Cell Signaling, 9754), H3K4me3 (Cell Signaling, 9751; active motif 39159), H3K27me3 (Cell Signaling, 9733), H3K9ac (Cell Signaling, 9649), H2AK119ub (Cell Signaling, 8240), rabbit polyclonal Ki67 (Vectorlabs), Lamin A/C (E-1), Lamin B1 (), PML (), hnRNPC1/C2 (Santa Cruz, SC-32308), DDX3 (Santa Cruz, SC-365768), DDX21 (Santa Cruz, SC-376953), Pol II (Santa Cruz, SC-47701), Caspase 3 (Cell Signaling, 9664), Caspase 7 (Cell Signaling, 8438), Caspase 8 (), PARP (SC-8007), actin (), SC35 (abcam, ab11826), hnRNPK (Santa Cruz, SC-28380), hnRNPU (Santa Cruz, SC-32315).

Immunoblotting and Immunofluorescence: as in [22, 56],

Crystal violet staining: Cells were cultured in 24-well plates to 60% confluency. Media then replaced with peptide supplemented Opti-MEM and incubated for the times indicated in the figures and legends. Later, cells were washed with PBS and fixed for 10 minutes in a 10% formalin solution. Cells were then rinsed with distilled water and subsequently stained with 100 *μ*l 0.1% crystal violet solution for 2 hours. Crystal violet-stained cells were observed and recorded using inverted optical microscopy [3].

Cell count analysis: Cells were plated in 6-well dishes and incubated in either 10μM of synthetic peptides in Opti-MEM reduced serum media or in 1 ml of complete growth media with puromycin antibiotic for the days indicated in the figures/legends. Subsequently cells were trypsinized and counted using a hemocytometer. This analysis is performed to study the effect of peptides on cell growth [22].

RNA isolation and reverse transcription–PCR analysis: Total RNA from peptide treated cells was prepared by using “RNeasy Mini Kit” (Qiagen, cat. No:74104) as per the manufacture’s protocol. RNA is converted to cDNA by using EcoDry Premix Double Primed (Clontech) kits. Quantitative RT-PCR was performed by using SsoFast Evagreen Supermix (Bio-Rad) as per the manufacturer’s protocol [57].

Generation of synthetic peptides: Peptides were synthesized at LifeTein at purity >75%. Peptides were dissolved in 100% DMSO at 1mg/ml and used at a concentration of 10μM in Opti-MEM media except where otherwise noted.

Annexin V-FITC and Propidium Iodide detection: We used the Annexin V-FITC Apoptosis Detection kit (ab14085) from Abcam. Cells were cultured on 24-well plates for 24 hours. The next day, media was replaced with fresh Opti-MEM containing 10μM peptide and further incubated for the times indicated. Subsequently, cells were washed once with PBS and subjected to the staining protocol according to the manufacturer’s instructions [3, 22].

Detection of Ki67 positive cells: Peptide treated cells were washed once with PBS and fixed in 4% PFA for 10 mins. Fixation was quenched with 125mM glycine in PBS for 10 mins. Cells were permeabilized with 0.25% triton x-100 in PBS for 10 mins followed by blocking buffer (5% FBS in PBS) for an additional 2 hr. Cells were incubated with Ki67-FITC antibody overnight in blocking buffer @1:1000 dilution. Cells were washed 3 times with PBS. Finally, to visualize the nuclei, cells were incubated with 1:10,000 diluted Hoechst in PBS for 15 mins. Ki67 signals were visualized and recorded under confocal microscopy [22].

CellTiter 96 Aqueous Cell proliferation (MTS) assay (Cat.No: G3582, Promega), CellTiter-Blue Cell viability Assay (Cat.No: G8080, Promega), RealTime-Glo MT Cell Viability Assay (Cat.No: G9711), CellTiter-Glo 2.0 Cell Viability Assay (Cat.No: G9241) were employed as per the methods recommended by the manufacturer.

Trypan Blue exclusion assay to determine the percentage of viable cells: Peptide treated cells were trypsinized and collected in 1ml of DMEM media. Cell suspension was mixed 1:1 ratio with 0.4% trypan blue solution. Non-viable cells are blue and viable cells are unstained. % viable cells were measured using a hemocytometer.

RT-PCR primer sequences: will be provided upon request.

## Supporting information

S1 Fig Viral-mediated expression of 9 randomly selected peptides in human fibroblasts. Representative anti-His immunofluorescence images of HFFs showing the viral expression of 9 randomly selected 15mer peptides. Scale bar, 10 μm.
(TIF)

S2 Fig A subset of random peptides differentially decrease cell viability in MDA-MB231 cells relative to primary mammary epithelial cells. A, B) Representative light microscopic images of crystal violet-stained MDA-MB231 (A) and PME (B) cells after 4 days of incubation in puromycin selection media. Red circles in A highlight peptide with differential effects in MDA-MB231 cells. C) Quantification of luminescence (RLU) of MDA-MB231 cells after 4 days of incubation in puromycin selection media. Y-Axis indicates the RLU; X-Axis indicates the PA peptide clone introduced by lentivirus transduction. Red bars highlight peptides statistically different from the unaffected peptide (PA46). For this analysis we considered PA46 as the internal control peptide as it behaved same as that of negative control peptides or untreated cells (data not shown). D) Sequential quantification of total cell number of MDA-MB231 cells at indicated days in Puromycin selection media after lentiviral transduction of individual peptide clones. Each line graph corresponds to a different virally expressed 15mer peptide. Red asterisks highlight peptides with differential effects in MDA-MB231 cells.
(TIF)

S3 Fig A) Representative anti-his immunofluorescence signal of HFFs cells treated with synthetic peptides for 4hr. The left panel represents Alexa Fluor 596-anti-his signal (red), the middle panel is Hoechst (blue), and the right panel corresponds to the merged image. Scale bar, 10 μm. B) Immunofluorescent detection of Ki67^+^ MDA-MB231 cells after 24hr of peptide treatment. Scale bar, 50 μm.
(TIF)

S4 Fig IC50 curves for cell viability at 48 hours of peptide treatment. (TIF)

S5 Fig Representative immunofluorescence for Caspase 3 (A), 7 (B) and PARP (C) in Con, PA10, PA15, PA38 and PA49 treated HCC11806 cells. Scale bar, 10 μm.
(TIF)

S6 Fig Western blots assaying for cleaved Caspase 3, cleaved Caspase 8, cleaved PARP and actin loading control in response to control and test peptides. (TIF)

S7 Fig Representative immunofluorescence images for lamin B1 (A), lamin A/C (B) and PML bodies (C) (red), nuclei (Hoechst, blue) in HCC1806 cells taken after 24hr of treatment with 10 μM peptide. Scale bar for A and B is 10 μm and C is 5 μm. Peptides are indicated at left.
(TIF)

S8 Fig Immunofluorescence for SC35 (A), DDX3 (B), DDX21 (C) and hnRNPK (D) (red); nuclei (Hoechst, blue) in peptide treated HCC1806 cells. Scale bars, 10 μm.
(TIF)

S1 Table Details of the 15mer random peptide sequences cloned in pLenti-puro vector and effects of their expression on HFFs, PME and MDA-MB231 cells. (XLSX)

S2 Table Viability of breast cancer and normal cell types in response to peptide treatment for 48 hours. Red bars on graphs indicate p value less than 0.01. (XLSX)

S3 Table Quantitation of Ki67+ cells in control and peptide treated HCC1806 cells at 24 hours. (XLSX)

S4 Table Quantitation of Annexin V+ cells in control and test peptide treated HCC1806 cells at 24 hours. (XLSX)

S5 Table Quantitation of Propidium Iodide + cells in control and test peptide treated HCC1806 cells at 24 hours. (XLSX)
