# Supplementary figures and images for: Discovery and characterization of anti-cancer peptides from a random peptide library
Source: PLoS One. 2024 Feb 13;19(2):e0293072. doi: 10.1371/journal.pone.0293072 (PMC10863893; doi:10.1371/journal.pone.0293072)

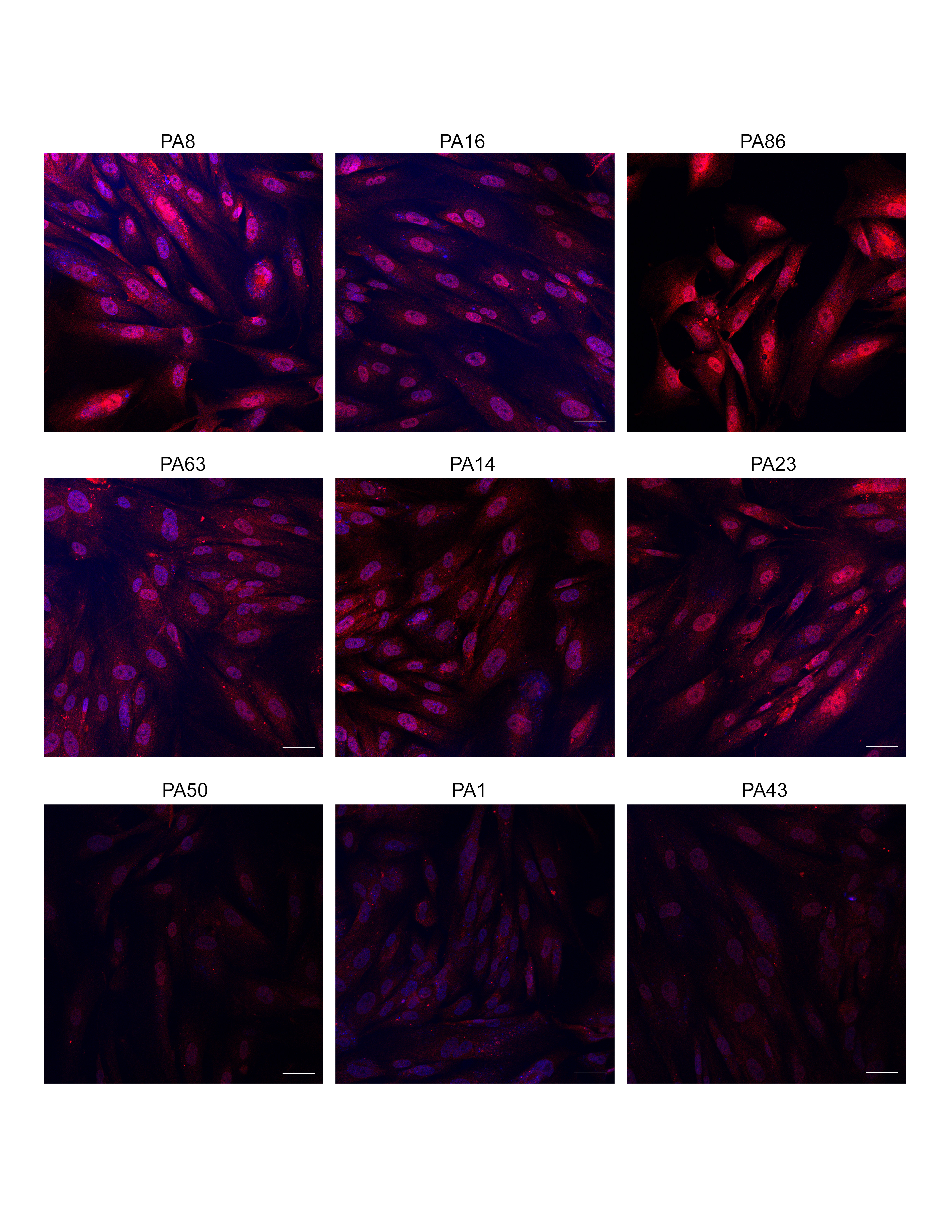

Supplement: S1 Fig — Representative anti-His immunofluorescence images of HFFs showing the viral expression of 9 randomly selected 15mer peptides. Scale bar, 10 μm. (TIF) [file pone.0293072.s001.tif]

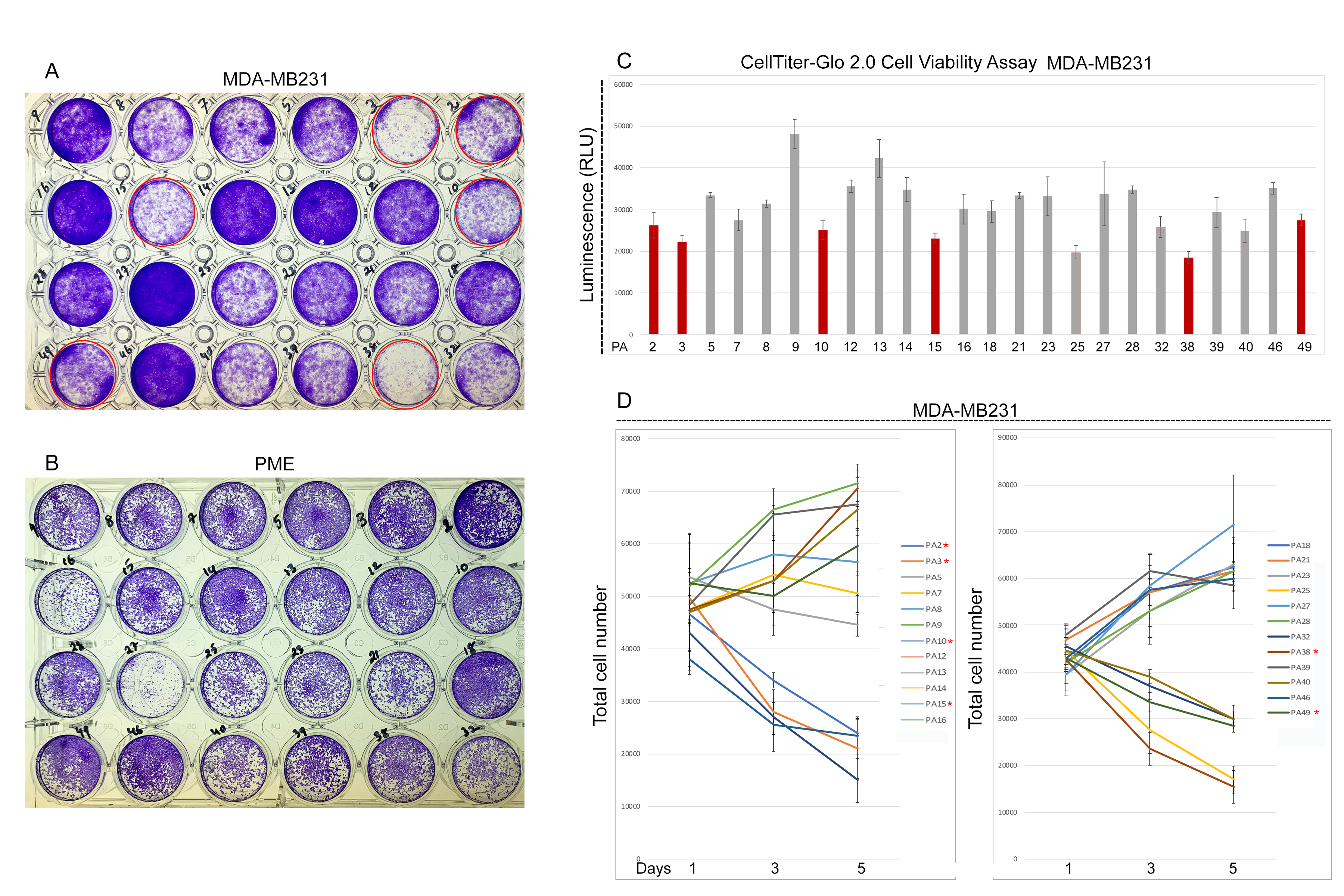

Supplement: S2 Fig — A, B) Representative light microscopic images of crystal violet-stained MDA-MB231 (A) and PME (B) cells after 4 days of incubation in puromycin selection media. Red circles in A highlight peptide with differential effects in MDA-MB231 cells. C) Quantification of luminescence (RLU) of MDA-MB231 cells after 4 days of incubation in puromycin selection media. Y-Axis indicates the RLU; X-Axis indicates the PA peptide clone introduced by lentivirus transduction. Red bars highlight peptides statistically different from the unaffected peptide (PA46). For this analysis we considered PA46 as the internal control peptide as it behaved same as that of negative control peptides or untreated cells (data not shown). D) Sequential quantification of total cell number of MDA-MB231 cells at indicated days in Puromycin selection media after lentiviral transduction of individual peptide clones. Each line graph corresponds to a different virally expressed 15mer peptide. Red asterisks highlight peptides with differential effects in MDA-MB231 cells. (TIF) [file pone.0293072.s002.tif]

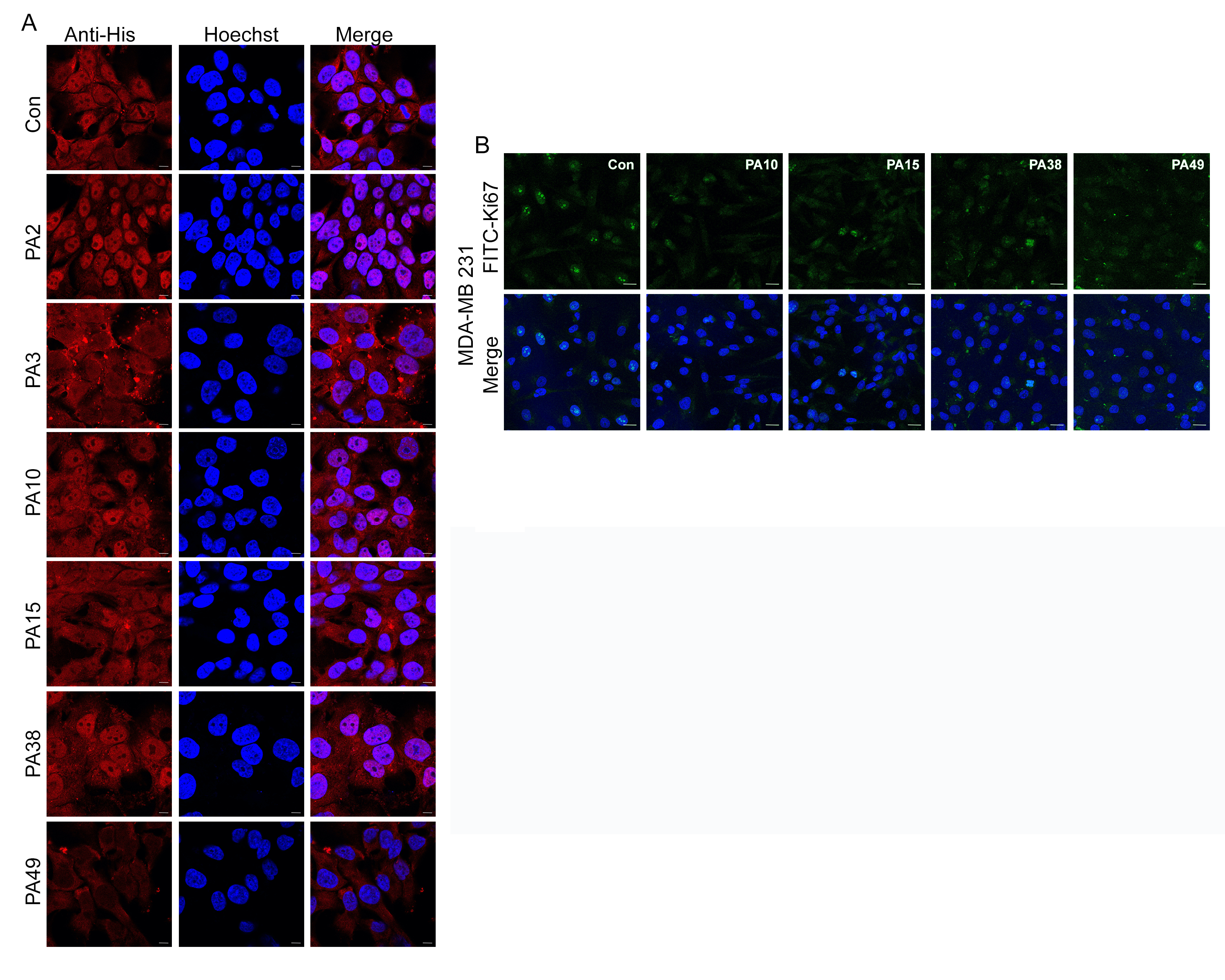

Supplement: S3 Fig — A) Representative anti-his immunofluorescence signal of HFFs cells treated with synthetic peptides for 4hr. The left panel represents Alexa Fluor 596-anti-his signal (red), the middle panel is Hoechst (blue), and the right panel corresponds to the merged image. Scale bar, 10 μm. B) Immunofluorescent detection of Ki67+ MDA-MB231 cells after 24hr of peptide treatment. Scale bar, 50 μm. (TIF) [file pone.0293072.s003.tif]

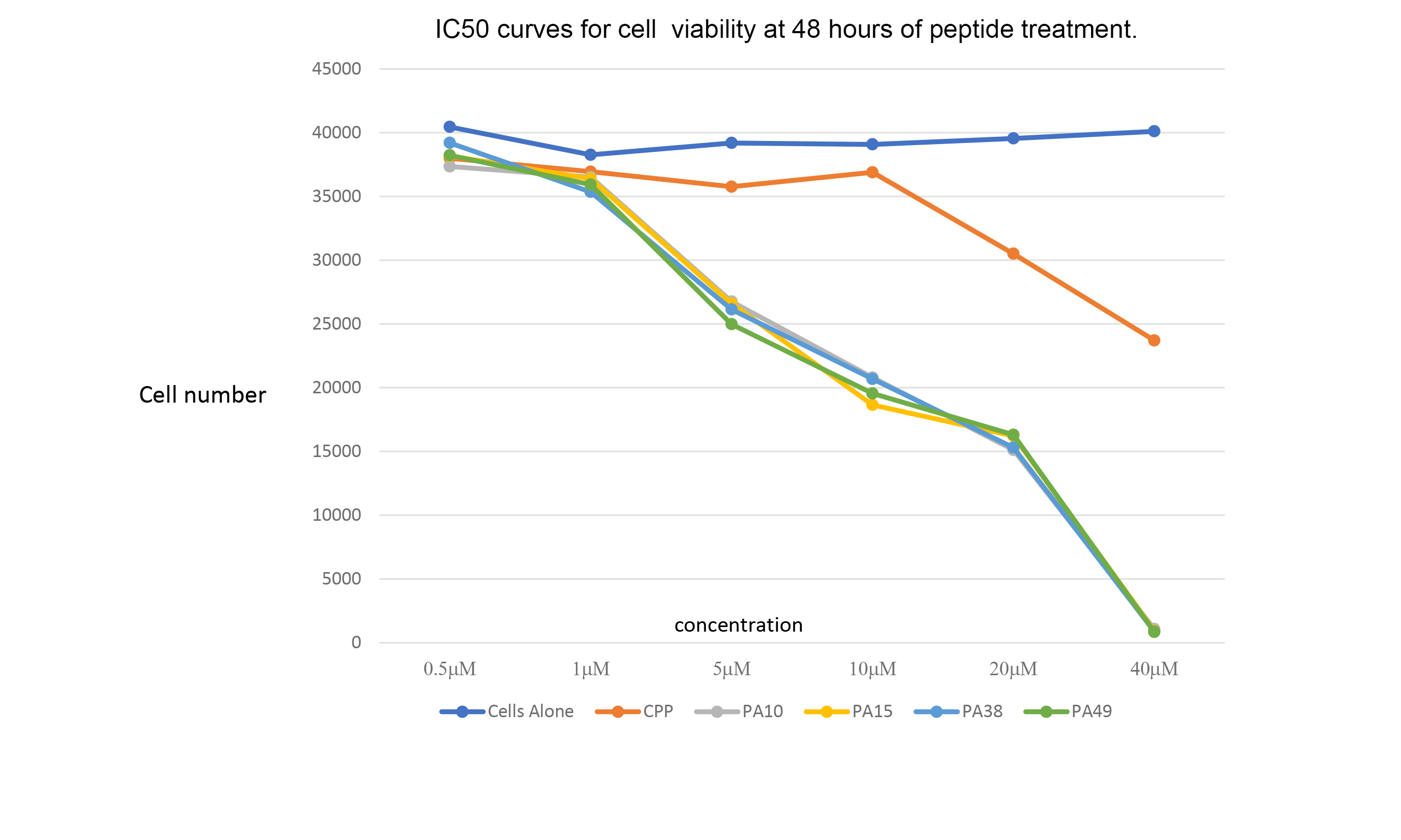

Supplement: S4 Fig — (TIF) [file pone.0293072.s004.tif]

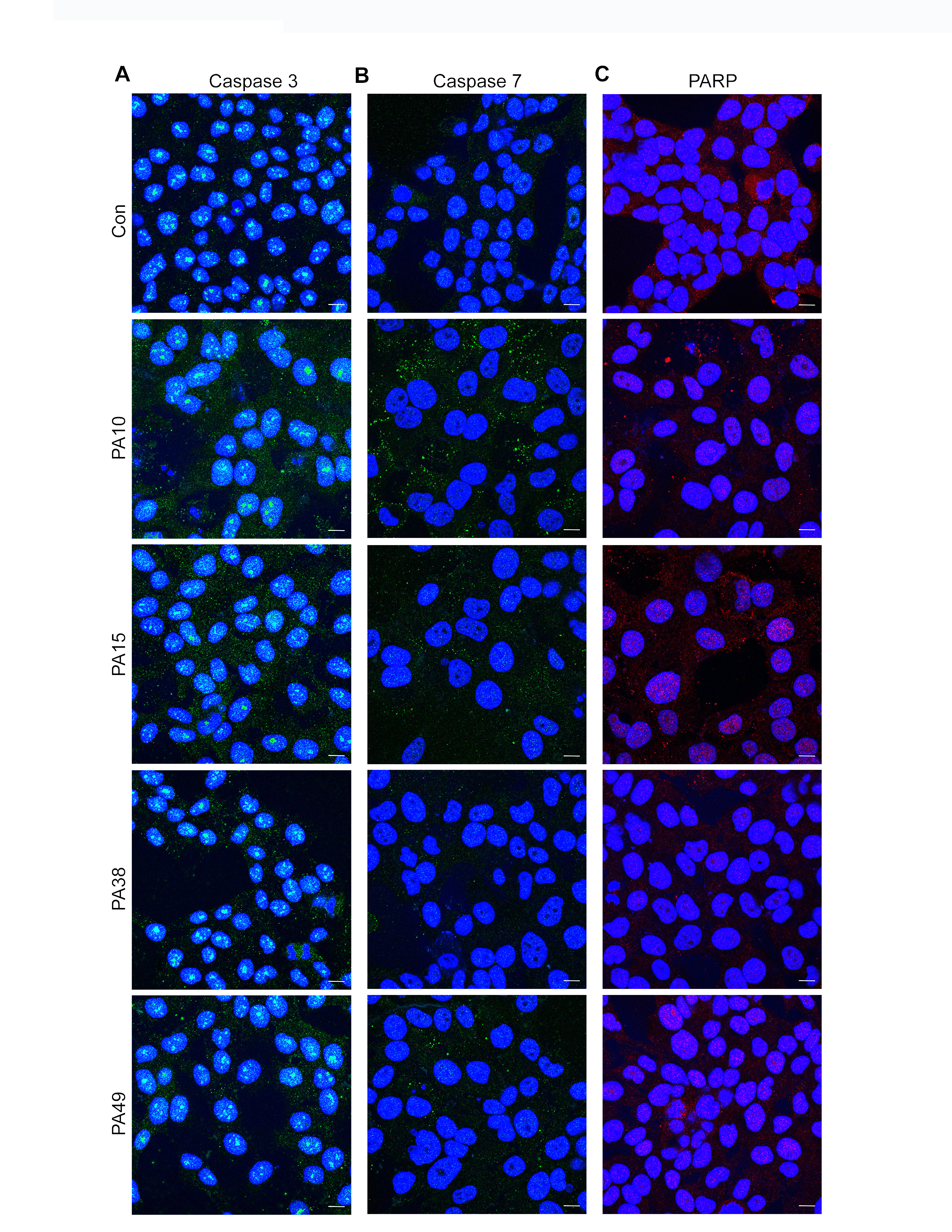

Supplement: S5 Fig — Representative immunofluorescence for Caspase 3 (A), 7 (B) and PARP (C) in Con, PA10, PA15, PA38 and PA49 treated HCC11806 cells. Scale bar, 10 μm. (TIF) [file pone.0293072.s005.tif]

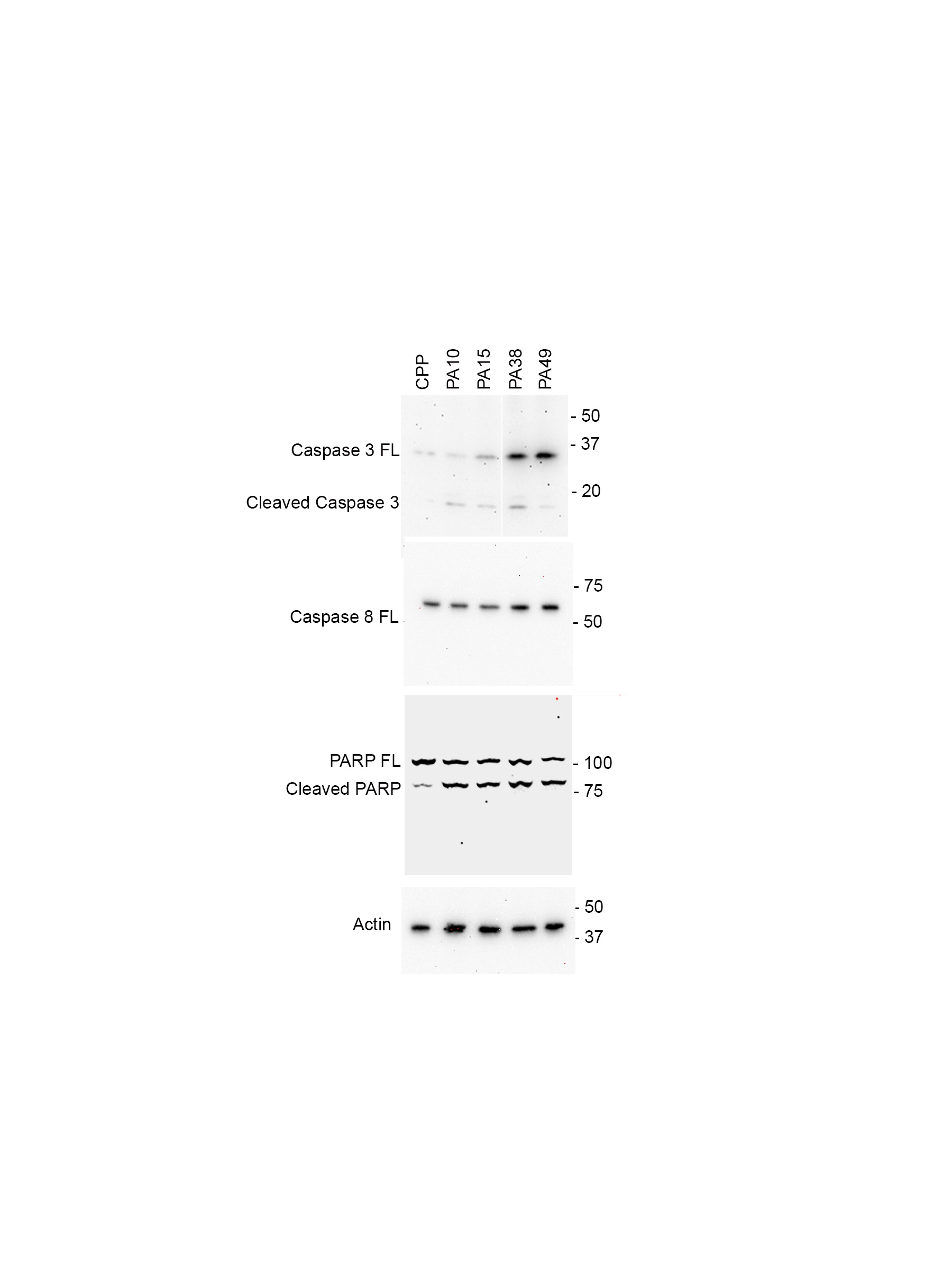

Supplement: S6 Fig — (TIF) [file pone.0293072.s006.tif]

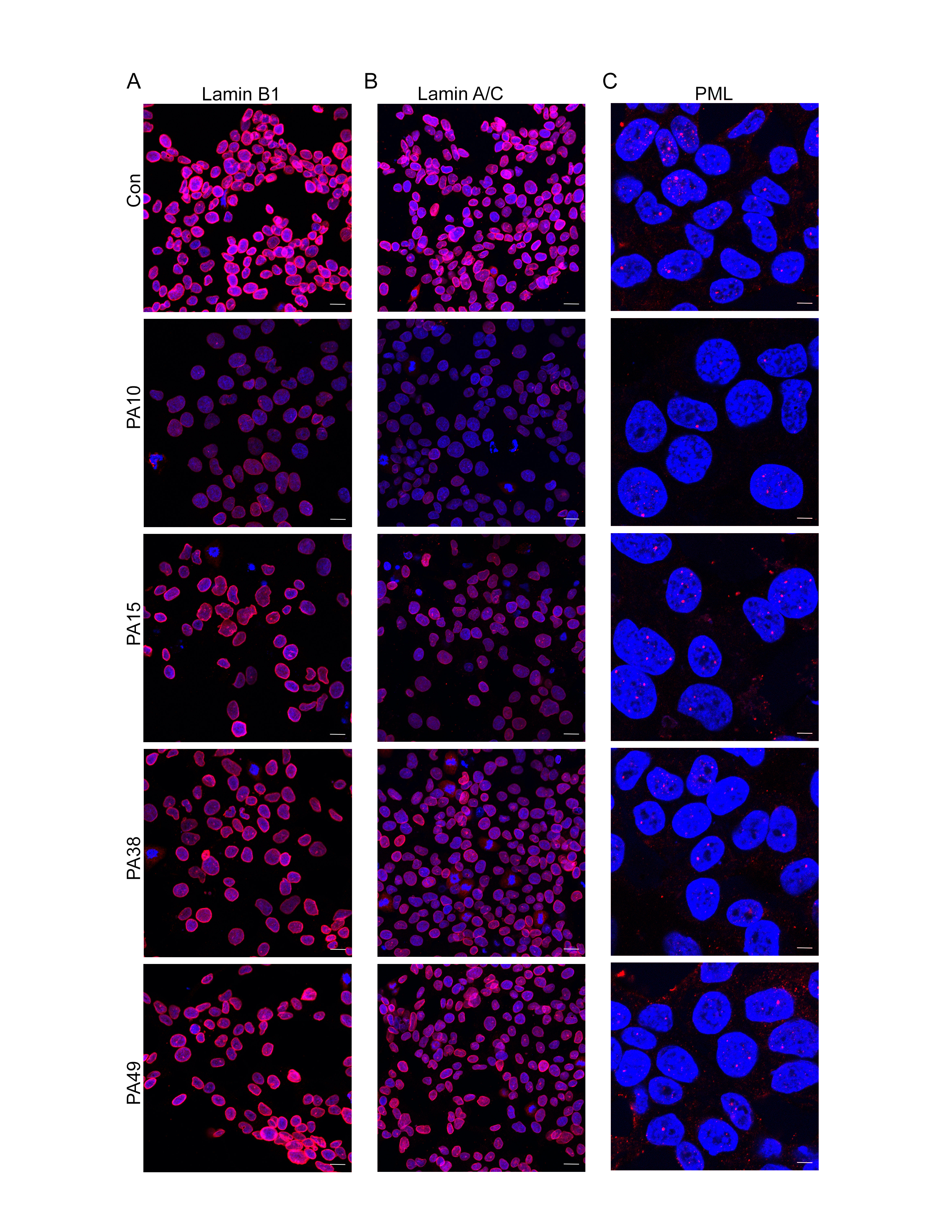

Supplement: S7 Fig — Representative immunofluorescence images for lamin B1 (A), lamin A/C (B) and PML bodies (C) (red), nuclei (Hoechst, blue) in HCC1806 cells taken after 24hr of treatment with 10 μM peptide. Scale bar for A and B is 10 μm and C is 5 μm. Peptides are indicated at left. (TIF) [file pone.0293072.s007.tif]

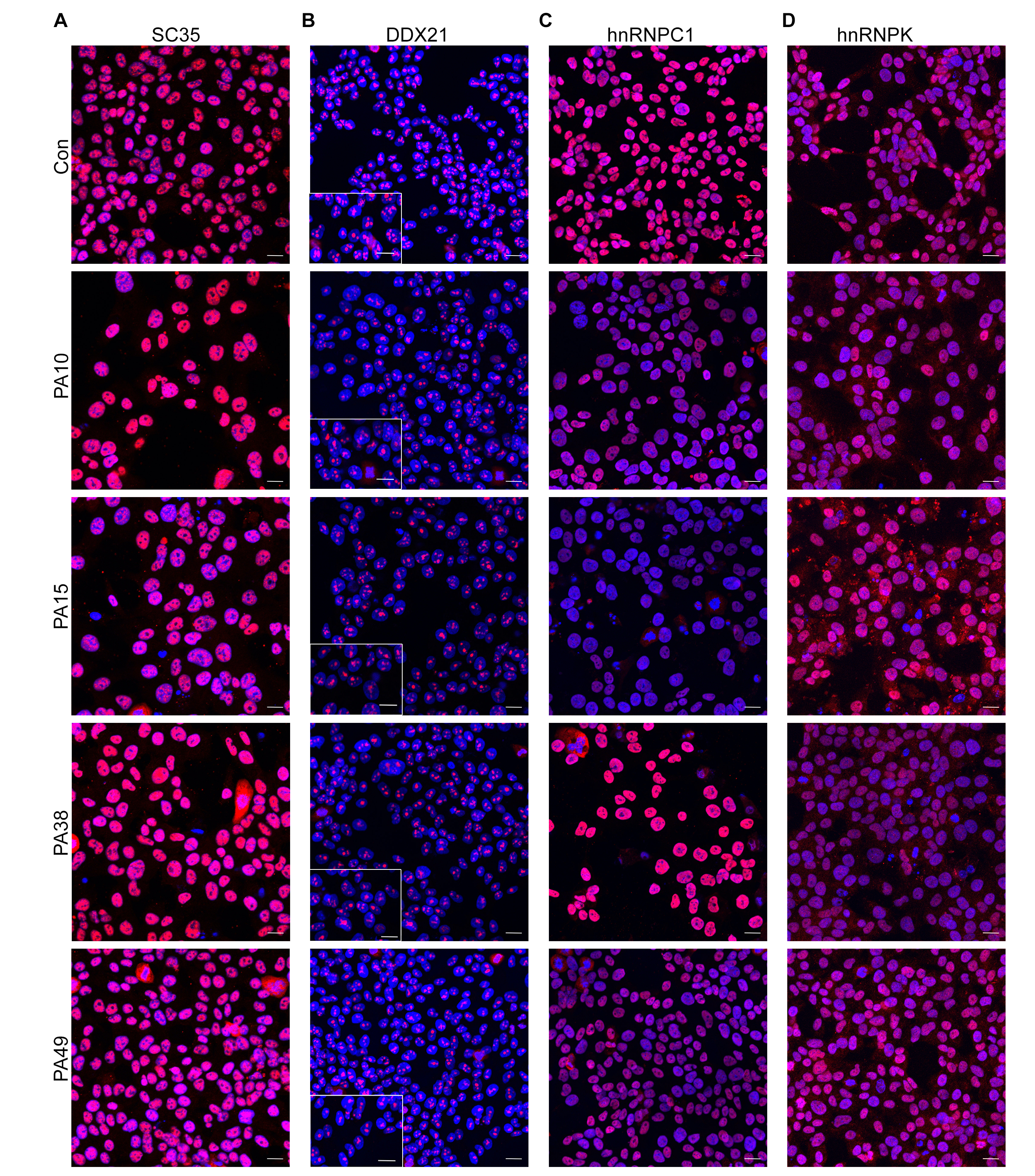

Supplement: S8 Fig — Immunofluorescence for SC35 (A), DDX3 (B), DDX21 (C) and hnRNPK (D) (red); nuclei (Hoechst, blue) in peptide treated HCC1806 cells. Scale bars, 10 μm. (TIF) [file pone.0293072.s008.tif]
